# Falling behind: The role of student loans on forgoing healthcare

**DOI:** 10.1111/hsc.13573

**Published:** 2021-09-20

**Authors:** Michael Babula, Alp Idil Ersoy‐Babula

**Affiliations:** ^1^ Department of Humanities and Social Sciences Khalifa University of Science and Technology Abu Dhabi UAE; ^2^ Lincoln International Business School University of Lincoln Lincoln UK

**Keywords:** educational debt, general health, healthcare, mental health, student loans

## Abstract

An area for concern in public health research is the extent to which USA student loans are associated with poor health. This study's objective examines whether falling behind on student loans may compound ill‐health by deterring people from seeking healthcare. The results of this study confirm that borrowers behind or in collections on student loans are forgoing healthcare after self‐reporting general physical ill‐health. This study used the 2019 Survey of Household Economics and Decision‐making (SHED) questionnaire that measures the economic well‐being of USA households. There were 337 participants behind or in collections on student loans. The size effects for forgoing mental healthcare and seeing a specialist were observed. Respondents behind on student loans were more likely to forgo mental healthcare (adjusted odds ratio (AOR) = 1.66, 95% confidence interval [CI] = 1.18, 2.33) and seeing a doctor/specialist (AOR = 1.53, 95% CI = 1.13, 2.07) even when controlling for sociodemographic variables, health insurance, childcare and eldercare payments, and medical debt. The implications for enlarging access to healthcare among people behind on student loans are discussed.

**What is known about the topic?**

- USA student loans have been associated with general ill‐health.
- Public and private USA student loans are nearly impossible to discharge via bankruptcy.
- USA student loans cause people to forgo major life events.

**What this paper adds?**

- This study adds that falling behind on own student loans associates with forgoing healthcare and compounds ill‐health.
- 67.4% and 50.4% of borrowers behind or in collections on student loans report being unable to find sufficient work and had major medical expenses, respectively.
- The results support bankruptcy for USA student loans to improve healthcare access.

## 1 INTRODUCTION

### 1.1 Personal debt and health

One strain of research contends that certain debts are associated with poor health and that ill‐health may be compounded by borrowers forgoing healthcare. High financial debt relative to assets has been associated with higher diastolic blood pressure and lower self‐reported ratings for global health (Sweet et al., 2013). The possibility that debt could have a compounded effect on general ill‐health is an issue that was raised, and yet unexamined, in earlier studies on mortgage foreclosures. Pollack and Lynch (2009) state:

Medical conditions and bills may worsen the emotional and financial stress faced by households undergoing mortgage foreclosure, and this increased stress may in turn exacerbate ill‐health (and may discourage people from obtaining further necessary medical care) (p. 1838).

Pollack and Lynch's reasoning can be extended to other forms of debt, which raises an important question. Should some forms of debt be prioritised when first examining the existence of a compounded effect of debt on poor health? In USA mortgage foreclosures, the proceeds from the home's sale cancel out the debt unless the amount owed exceeds the amount received, creating a deficiency in certain states (see Demiroglu et al., 2014). The mortgage debtor can further file for bankruptcy, discharging the deficiency with a terminal date in court and eliminate the initial stressor. Most other forms of debt are also dischargeable via bankruptcy. For instance, more than 20% of addicted gamblers end up discharging their debt via bankruptcy (Fay, 2021).

However, USA private and public student loans are mainly non‐dischargeable via bankruptcy owing to the legal test established in *Brunner v New York State Higher Education Services Corp*. (1987). Student loans may subject borrowers facing difficulty to unrelenting stress as the debtor cannot envisage a way out. As a result, this study's approach is to prioritise the examination of a compounded effect of student loans. Acquiring a greater perspective of this phenomenon may play a role in modifying bankruptcy laws or reinterpreting existing laws to permit discharge and elimination of the stressor that is suspected to be contributing to the compounded effect on poor health.

### 1.2 Associations between student loans and ill‐health

During the COVID‐19 pandemic, there has been a temporary moratorium on student loan repayment. However, it is important to historically examine data before the COVID‐19 crisis to observe clues as to whether people will once again forgo healthcare when the brief moratorium offered on student debt repayment ends.

In 2019, 18% of all USA student loan borrowers were in default (Baum et al., 2019). Other loan borrowers may be falling behind on student loans but do not go into default and collections. A student loan becomes delinquent the first day after the borrower misses a payment (U.S. Department of Education, 2019). Many borrowers may experience the heightened stress of becoming regularly delinquent and forgo healthcare or other vital necessities to bring their loans current. It is also important to mention that the 18% default figure does not account for home equity loans, credit cards and other debts students acquire along with student loans to pay educational expenses. In addition, the Kluender et al. (2021) study found that 17.8% of Americans had medical debts in collections before the COVID‐19 pandemic.

One might assume that student loans to finance education would be beneficial to health as they enhance access to higher education so that financially strained students have the chance to find personal meaning through academic achievement. The Herd (2010) study suggested that academic performance was strongly linked to better health later in life. However, it is important to delineate the health benefits of academic performance from the ill‐health effects of potentially unforgivable debt. The public health literature makes this distinction and indicates that student loans are often associated with ill‐health consequences. A consensus among social scientists is that student loans are linked to increases in stress, depression, suicide, increased psychological problems, and decreased life satisfaction (Kim & Chatterjee, 2019). In addition, the effect of student loans on ill‐health disproportionally affects racial and ethnic minorities. Black young adults with greater student loans reported shorter sleep duration when controlling for a series of confounding variables (Walsemann et al., 2016).

Walsemann et al. (2016) showed that student loans cause people to forgo expenses such as homeownership or delay marriage. Given the scholarship discussed, this study's objective is to explore whether there is a compounding effect for student loans on ill‐health where borrowers behind or in collections are forgoing healthcare.

## 2 METHODS

The Survey of Household Economics and Decision‐making (SHED) was used to address student loans, general physical health and forgoing medical care (see Canilang et al., 2020). The Federal Reserve Board's objective in using the SHED is to better “understand the wide range of financial challenges and opportunities facing families in the United States” (Canilang et al., 2020, p. 1). The SHED contains questions about borrowers (e.g., students and former students) to address this study's objective.

### 2.1 Participants

The SHED included non‐institutionalised adults aged 18 and over living in the USA (Canilang et al., 2020). The data revealed that no participants over 93 years of age completed the survey. Canilang et al. (2020) states, “Of the 19,994 participants contacted to take the 2019 SHED, 12,238 (excluding breakoffs) participated, yielding a final stage completion rate of 61.2 percent…The final sample used in the report included 12,173 respondents” (p. 57). It is further noted that “Of the 12,238 respondents who completed the survey, 65 were excluded from the analysis…due to either leaving the responses to a large number of questions missing, completing the survey too quickly, or both” (Canilang et al., 2020, p. 57). Overall, the sample included slightly more males (51.7%) than females (48.3%), and there were a higher number of married respondents (57.3%).

### 2.2 Procedure

The SHED data collection is conducted through Ipsos, a private consumer research firm that administered the survey using KnowledgePanel (Canilang et al., 2020). KnowledgePanel is a probability‐based online panel and is weighted to be representative of all adults living in the USA (Canilang et al., 2020). Ipsos recruited voluntary respondents through an address‐based sampling methodology (ABS) because this method is considered representative of the population (Board of Governors of the Federal Reserve System, 2020). The KnowledgePanel was weighted “to the benchmarks in the 2019 March supplement of the Current Population Survey” so that the sample matched that of U.S. adults (Canilang et al., 2020, p. 18). Canilang et al. specify that “The geo‐demographic dimensions used for weighting the entire KnowledgePanel included gender, age, race, ethnicity, education, census region, household income, homeownership status and metropolitan area status” (p. 18). The survey was conducted from October 11, 2019, until October 24, 2019, and on average, the survey took 19 min (median time) to complete (Canilang et al., 2020).

### 2.3 Survey of household economics and decision‐making measure

Larrimore et al. (2015) compared identical and similar SHED questions and datasets to the Census Bureau's Current Population Survey (CPS), American Community Service (ACS), and Survey of Income and Program Participation (SIPP) and found the SHED to be consistent with validity and reliability estimates from other datasets.

Student loans were assessed using the question, “Are you behind on payments or in collections for one or more of the loans from your own education?” The categories for this question were coded 0 = no, 1 = yes. The general health categories for the question, “In general, would you say your physical health is…” (1 = excellent, 2 = very good, 3 = good, 4 = fair, and 5 = poor) were recoded 0 = good, 1 = poor. Items for forgoing healthcare were assessed by five single‐item measures assessing respondents' need to forgo the item in the past 12 months because the respondent could not afford it. These included (a) mental healthcare or counselling, (b) seeing a doctor or specialist, (c) follow‐up care, (d) prescription medicine, and (e) dental care. The response categories were coded 0 = did not forgo healthcare, 1 = forwent healthcare.

Additional items associated with forgoing healthcare include insurance coverage. Six single items for insurance (e.g., employer or union, insurance company, Medicare or Medicaid, military or veterans care, health insurance exchange and other forms of insurance) were coded as dummy variables and summed into a single item and recoded 0 = uninsured, 1 = insured. Categories for paying for childcare or eldercare included (0 = no, 1 = yes); debt from unexpected medical expenses in the past 12 months were included (0 = no, 1 = yes).

### 2.4 Demographic question items

Participants reported their gender (1 = male, 2 = female), and children under 18 years‐old living with the respondent (1 = yes, 2 = no). Age was assessed numerically. Some data transformation took place with demographic items to facilitate data analysis. Marital status categories (1 = married, 2 = widowed, 3 = divorced, 4 = separated, 5 = never married and 6 = living with partner) were recoded as 1 = married, 2 = not married. The race and ethnicity categories (1 = white, 2 = black, 3 = other, 4 = Hispanic) were recoded 1 = white, 2 = black and 3 = other/Hispanic. Educational categories (1 = less than a high school degree, 2 = high school, 3 = some college, but no degree, 4 = certificate or technical college, 5 = associate's degree, 6 = bachelor's degree and 7 = graduate degree) were recoded 1 = high school or less, 2 = some college, and 3 = bachelor's degree or higher. Employment status categories (1 = working as a paid employee, 2 = working as self‐employed, 3 = not working on a temporary layoff from a job, 4 = not working looking for work, 5 = not working retired, 6 = not working disabled and 7 = not working other) were recoded 1 = employed, 2 = not working. Household income categories include 1 through 11 ≤ $49,999, 12 through 21 ≥ $50,000, and were recoded 1 < 200% federal poverty line (FPL) for a family of four, 2 > 200% FPL for a family of four as the original variable's categories approximate the FPL (see Assistant Secretary for Planning & Evaluation, 2019 for FPL calculations).

### 2.5 Data analysis plan

Many of the variables on the SHED are categorical. For this reason, analysis was limited to primarily *χ* ^2^ to analyse sociodemographic items and participants falling behind or going into collections on student loans. A *t*‐test test was used to explore differences in age and lateness in paying student loan obligations. Three binary logistic regression models were formed where forgoing different types of healthcare were entered as dependent variables and falling behind or going into collections on student loans was entered as the independent variable.

The variables were added to the models based on patterns observed in the existing literature. Demographic items such as gender, age and income have been associated with forgoing mental healthcare, and these variables were added to the first model (Andrade et al., 2014; Sareen et al., 2007). The Pollack and Lynch (2009) study added socioeconomic indicators such as education level, whether household income was less than 200% of the federal poverty level, and participants' employment status when evaluating the health impacts of home foreclosures on individuals. These variables were added to the second logistic regression model. The literature also indicates that health insurance coverage, childcare and medical debt should be controlled when analysing either ill‐health or healthcare access (Ahmed et al., 2001; Kim & Chatterjee, 2019). These variables were added as adjustments to the third model. Overall, the objective was to observe if these predictors would interact to mitigate the effects of student loans in predicting participants' decisions to forgo healthcare.

### 2.6 Ethical considerations

The USA executive branch's Office of Management and Budget (OMB) (2020) certified that the SHED (FR 3077; OMB control number 7100‐0374) complies with 5 CFR 1,320.9 and the related provisions of 5 CFR 1,320.8(b)(3) that inform respondents why information is being collected and how it will be used. The Board of Governors of the Federal Reserve System (2020) stipulates that the SHED is voluntary and that personally identifiable information is withheld under exemption 6 of the Freedom of Information Act.

## 3 RESULTS

### 3.1 Demographic analysis of participants' student loan status

There were 337 participants behind on payments or in collections for one or more of their student loans compared with 1,582 current on student loans. Table 1 provides the demographic characteristics for this sample. Individuals who were significantly more likely to be behind or in collections on student loans are described as older, unmarried, members of racial and ethnic minorities, respondents under the federal poverty line, unemployed, and those with less than a bachelor's degree.

**TABLE 1:** Demographic characteristics of participants behind or in collection on student loans and the total sample

	Participants behind on payments or in collections on student loans, No. (%) or Mean	Participants current on student loans, No. (%), or Mean	*p* ^a^
Total number, no. (%)	337 (17.6)	1,582 (82.4)	
Gender, no. (%)			0.518
Male	135 (40.1)	664 (42.0)	
Female	202 (59.9)	918 (58.0)	
Age, y, mean	38.91	35.12	0.000
Race/ethnicity, no. (%)			0.000
White	134 (39.8)	957 (60.5)	
Black	105 (31.2)	262 (16.6)	
Other/Hispanic	98 (29.1)	363 (22.9)	
Education level, no. (%)			0.000
Bachelor's degree or higher	97 (28.8)	985 (62.3)	
Some college	167 (49.6)	479 (30.3)	
High school or less	73 (21.7)	118 (7.5)	
Marital status, no. (%)			0.018
Married	116 (34.4)	655 (41.4)	
Unmarried	221 (65.6)	927 (58.6)	
Children under age 18 in household, no. (%)			0.003
Yes	147 (44.0)	557 (35.3)	
No	187 (56.0)	1,019 (64.7)	
Employment Status, no. (%)			0.000
Employed	248 (73.6)	1,354 (85.6)	
Unemployed	89 (26.4)	228 (14.4)	
Income <200% FPL for a family of four, no. (%)			0.000
Yes	213 (63.2)	514 (32.5)	
No	124 (36.8)	1,068 (67.5)	

Note

Data are from the 2019 Survey of Household Economics and Decision‐making (SHED).

Abbreviation: FPL, federal poverty line.

^a^ Significance for the *χ* ^2^ test for categorical variables examining significant differences between participants who have fallen behind or are in collections on their student loans and those who are not, *t*‐test was used for the continuous variable (age).

The type of educational debt that most participants reported is 95.9% student loans. These borrowers often have other forms of educational debt along with student loans: 3.6% are home equity loans, 23.0% are credit card loans and 10.9% are other types of loans. Although the SHED data were limited in directly asking why students were behind on student loans, contingency tables provided some insight. Of those behind or in collections on student loans, 67.4% of participants behind on student loans reported finding insufficient work compared with 42.5% who were current; 50.4% had major medical expenses compared with 24.4% who were current; 12.1% self‐report temporary employment compared with 7.4% who were current; and 9.2% of respondents had been fired or laid off in the past 12 months compared with 3.6% who were current.

### 3.2 Compounding effect of student loans on forgoing healthcare

Table 2 indicates that participants who were behind or in collections on student loans are significantly more likely to forgo mental healthcare and counselling, seeing a doctor or specialist, follow‐up care, prescriptions and dental care when adjusting for sociodemographic variables, childcare and eldercare expenses, health insurance and medical debt.

**TABLE 2:** Logistic regression analysis predicting forgoing healthcare from being behind or in collections on own student loans

During the past 12 months, was there a time when you needed any of the following but went without because you could not afford it?	Participants reporting that they are behind or in collections on student loans, No (% within the healthcare category)	Participants current on student loans, No (% within the healthcare category)	Sig^a^	Model 1, exp b^b^ (95% CI)	Sig	Model 2, exp b^b^ (95% CI)	Sig	Model 3 exp b^b^ (95% CI)	Sig
Mental healthcare or counselling	84 (25.9)	249 (15.7)	0.000	2.33 (1.72, 3.16)	0.000	2.12 (1.54, 2.92)	0.000	1.66 (1.18, 2.33)	0.004
Seeing a doctor/specialist	135 (27.8)	201 (14.1)	0.000	2.46 (1.90, 3.20)	0.000	2.09 (1.59, 2.75)	0.000	1.53 (1.13, 2.07)	0.006
Follow‐up care	104 (35.5)	230 (14.2)	0.000	3.08 (2.31, 4.12)	0.000	2.51 (1.86, 3.40)	0.000	1.90 (1.37, 2.63)	0.000
Prescription medicine	103 (35.4)	231 (14.3)	0.000	3.22 (2.41, 4.29)	0.000	2.51 (1.85, 3.39)	0.000	1.95 (1.42, 2.69)	0.000
Dental care	172 (30.7)	164 (12.1)	0.000	3.09 (2.41, 3.98)	0.000	2.39 (1.84, 3.11)	0.000	1.89 (1.43, 2.51)	0.000

^a^ Significance level for *χ* ^2^ that explored association between being behind or in collections on student loans and forgoing healthcare.

^b^ The predicted change in the odds ratio, CI, confidence interval (rounded up to two decimal places). Model 1 was adjusted for age, gender, marital status, and race and ethnicity. Model 2 was adjusted for age, gender, marital status, race and ethnicity, education, income approximately below 200% of the federal poverty level, and unemployment status. Model 3 was adjusted for age, gender, marital status, race and ethnicity, education, income below 200% of the federal poverty level, unemployment status, child or elder care payments, health insurance coverage and medical debts.

A logistic regression model was run to predict general physical health from falling behind or going into collections on student debt while controlling for demographic variables used as predictors in model three in Table 2. Participants behind on student loans were significantly more likely to self‐report poor general physical health (AOR = 1.52, CI = 1.07, 2.15, *p* < 0.019).

## 4 DISCUSSION

The data demonstrates that falling behind or going into collections on student loans are significantly associated with forgoing mental healthcare and counselling, seeing a doctor or specialist, follow‐up care, prescriptions and dental care when adjusted for confounding variables. Participants also self‐reported poorer general physical health associated with falling behind or going into collections on student loans. USA student loans can last years or even a person's lifespan and are generally not dischargeable in bankruptcy, creating a cycle of contributing to general ill‐health while impeding public health goals of treating and managing illnesses. It is important to break down the causes of inaccessible healthcare for those having trouble paying student loans if medical professionals are to help break the cycle.

One surface‐level assumption is that individuals are falling behind on student loans because of high medical costs. There is some truth to the claim that medical expenses are heightening student loan delinquency and default. Medical expenditures were the second leading cause of why participants fell behind or went into collections on student loans. Elliott and Nam (2013) indicated that households with student loans have lower net worth, and by logical extension, lower savings to cover student loans if unexpected medical expenses arise. In addition, Minicozzi (2005) showed that incrementally higher student loans result in lower wage growth four years after people finish college, demonstrating further evidence of lower disposable income to afford student loans should ill‐health and associated medical costs arise after acquiring the debt.

However, similar to the Pollack and Lynch (2009) research about mortgage foreclosures, the data here indicate that causality is bidirectional and inaccessibility to certain forms of healthcare and general ill physical health are consequences of falling behind on student loans. Lazarus and Folkman (1984) state that “psychological stress is a particular relationship between the person and the environment that is appraised by the person as taxing or exceeding his or her resources and endangering his or her well‐being” (p. 19). Student loans can place significant stress on people that may overwhelm an individual's time and decision‐making resources in obtaining healthcare. Stress associated with debt was highlighted in research on mortgage foreclosures with a terminal date in court (Pollack & Lynch, 2009). For several reasons, the stress associated with student loans may be worse given the harsh consequences of student loan default.

For example, the stress produced from defaulting on a student loan can result from garnishment to wages, Social Security disability and retirement benefits (U.S. Department of Education, 2018). The U.S. Department of Education also indicates that people in default are at risk of losing their driver's license and other state‐issued licenses, preventing them from travelling to work or seeking adequate full‐time employment. Even for borrowers and co‐signers who are not in default, individuals holding substantial student loans will find that credit scores are affected, making it difficult to obtain mortgages or other major purchases (Elliott & Nam, 2013).

The focal point for this discussion has so far focussed on how student loans may heighten stress levels among ordinary borrowers, but try to imagine the stress produced by student loans for chronically ill individuals. Woolf and Aron (2013) indicate that people often experience stress related to disorganised healthcare, such as delays, duplication of medical tests and inaccessible after‐hours medical services. A combination of stressors in the form of student loans and inefficient healthcare systems is more than enough to overwhelm a person's coping mechanisms and lead people to place less emphasis on seeking certain healthcare services. The most worrisome part of this research is that participants were forgoing mental healthcare and counselling which could help mitigate stress and lead to referrals for comorbid medical conditions. The healthcare profession operates best through an interlocking chain of cooperation, but student loans affect people in a way that prevents maximised contact with a spectrum of medical professionals and results in an unnecessary detriment to vulnerable individuals that healthcare is supposed to serve.

Policy makers may need to become more innovative to resolve the student loan crisis and its impact on accessing healthcare. Most universities offer exit counselling to students about to graduate, but many counsellors have not been trained to advise students on obtaining public health assistance should they fall behind on student loan payments. Pollack and Lynch (2009) have recommended placing counsellors at mortgage counselling centres to provide advice to help individuals locate community safety nets. By parity of reasoning, crisis counsellors should be placed at university financial aid offices and student debt collection agencies. Like other forms of debt, student loans disproportionally affect racial and ethnic minorities and those under 200% of the federal poverty line, groups that traditionally face barriers to accessing healthcare. It may also be necessary to triage individuals to help the most vulnerable achieve timely access to mental healthcare and counselling, seeing a specialist, follow‐up care, prescription medications and dental care.

The phenomenon of forgoing healthcare owing to student loans was observed in a strong economy before the COVID‐19 crisis. Although the USA provided a brief moratorium on student loans, unemployment levels have increased, and current and future borrowers are likely to be in a much worse financial situation when forbearance ends. Student loans coupled with high unemployment may impact individuals' ability to obtain healthcare where an estimated 3.3 million Americans, and increasing, have contracted a debilitating form of long COVID that may affect the ability to work (Hart, 2021). The government could offer to subsidise healthcare for individuals falling behind on student debt, and while this option might open a gateway to make certain forms of healthcare more accessible, it is not likely to remove the underlying stress and general ill‐health associated with student loans. The more comprehensive option that has been debated for decades, but has received little legislative support, is to reconsider making private and public student loans dischargeable via bankruptcy proceedings.

### 4.1 Limitations and future research

There will exist some selection bias in the SHED data because the survey is based on self‐reports, and memories are sometimes inaccurate. Although the scope of this research focused on student loans, other forms of educational debt (e.g., home loans, credit cards and loans from relatives) are often combined with student loans in financing education, but questions for encountering difficulties with other forms of educational debt were not included on the SHED. Also, the SHED does not include questions on how many payments or how long participants' fall behind on student loans. Future research should investigate these items and whether student loans combined with other forms of educational debt produce an additive compounded effect on general ill‐health.

## CONFLICT OF INTEREST

The authors declare that they have no conflict of interest.

## Data Availability

The data that support the findings of this study are openly available in the Board of Governors of the Federal Reserve System repository at https://www.federalreserve.gov/consumerscommunities/shed_data.htm

## References

[hsc13573-bib-0001] Ahmed, S. M. , Lemkau, J. P. , Nealeigh, N. , & Mann, B. (2001). Barriers to healthcare access in a non‐elderly urban poor American population. Health & Social Care in the Community, 9(6), 445–453. 10.1046/j.1365-2524.2001.00318.x 11846824

[hsc13573-bib-0002] Andrade, L. H. , Alonso, J. , Mneimneh, Z. , Wells, J. E. , Al‐Hamzawi, A. , Borges, G. , Bromet, E. , Bruffaerts, R. , de Girolamo, G. , de Graaf, R. , Florescu, S. , Gureje, O. , Hinkov, H. R. , Hu, C. , Huang, Y. , Hwang, I. , Jin, R. , Karam, E. G. , Kovess‐Masfety, V. , … Kessler, R. C. (2014). Barriers to mental health treatment: Results from the WHO World Mental Health surveys. Psychological Medicine, 44(6), 1303–1317. 10.1017/S0033291713001943 23931656 PMC4100460

[hsc13573-bib-0003] Assistant Secretary for Planning and Evaluation . (2019). 2019 poverty guidelines. Accessed July 20, 2021. https://aspe.hhs.gov/topics/poverty‐economic‐mobility/poverty‐guidelines/prior‐hhs‐poverty‐guidelines‐federal‐register‐references/2019‐poverty‐guidelines

[hsc13573-bib-0026] Baum, S. , Ma, J. , Pender, M. , & Libassi, C. J. (2018). Trends in student aid. The college board. Accessed November 28, 2019. https://research.collegeboard.org/pdf/trends‐student‐aid‐2018‐full‐report.pdf

[hsc13573-bib-0004] Board of Governors of the Federal Reserve System . (2020). Supporting statement for the survey of household economics and decisionmaking. Accessed July 20, 2021. https://omb.report/icr/202010‐7100‐015/doc/105697301

[hsc13573-bib-0005] Brunner v New York State Higher Education Services Corp . (1987). 831 F.2d 395 (2d Cir.).

[hsc13573-bib-0006] Canilang, S. , Duchan, C. , Kreiss, K. , Larrimore, J. , Merry, E. , Troland, E. , & Zabek, M. (2020). Report on the economic well‐being of households in 2019. Accessed July 20, 2021. https://www.federalreserve.gov/publications/files/2019‐report‐economic‐well‐being‐us‐households‐202005.pdf

[hsc13573-bib-0007] Demiroglu, C. , Dudley, E. , & James, C. M. (2014). State foreclosure laws and the incidence of mortgage default. The Journal of Law and Economics, 57(1), 225–280. 10.1086/674868

[hsc13573-bib-0008] Elliott, W. , & Nam, I. (2013). Is student debt jeopardizing the short‐term financial health of U.S. households? Federal Reserve Bank of St. Louis Review, 95(5), 405–425. 10.20955/r.95.405-424

[hsc13573-bib-0009] Fay, M. (2021). Gambling and debt. Accessed August 25, 2021. https://www.debt.org/advice/gambling/

[hsc13573-bib-0010] Hart, R. (2021). Here's what we know about long Covid, the debilitating, lingering illness that could affect millions. Forbes. https://www.forbes.com/sites/roberthart/2021/07/09/heres‐what‐we‐know‐about‐long‐covid‐the‐debilitating‐lingering‐illness‐that‐could‐affect‐millions/?sh=69a8f607114e

[hsc13573-bib-0011] Herd, P. (2010). Education and health in late‐life among high school graduates: Cognitive versus psychological aspects of human capital. Journal of Health and Social Behavior, 51(4), 478–496. 10.1177/0022146510386796 21131622 PMC6339685

[hsc13573-bib-0012] Kim, J. , & Chatterjee, S. (2019). Student loans, health, and life satisfaction of USA households: Evidence from a panel study. Journal of Family and Economic Issues, 40(1), 36–50. 10.1007/s10834-018-9594-3

[hsc13573-bib-0013] Kluender, R. , Mahoney, N. , Wong, F. , & Yin, W. . (2021). Medical debt in the US, 2009‐2020. JAMA, 326(3), 250–256. 10.1001/jama.2021.8694 34283184 PMC8293024

[hsc13573-bib-0014] Larrimore, J. , Schmeiser, M. D. , & Devlin‐Foltz, S. (2015). Should you trust things you hear online? Comparing SHED and Census Bureau survey results. FEDS Notes. Accessed October 15, 2015. 10.17016/2380-7172.1619

[hsc13573-bib-0015] Lazarus, R. S. , & Folkman, S. (1984). Stress, appraisal, and coping. Springer Publishing Company.

[hsc13573-bib-0016] Minicozzi, A. (2005). The short term effect of educational debt on job decisions. Economics of Education Review, 24(4), 417–430. 10.1016/j.econedurev.2004.05.008

[hsc13573-bib-0017] Office of Management and Budget . (2020). Survey of household economics and decisionmaking. Accessed July 20, 2021. https://omb.report/icr/202010‐7100‐015

[hsc13573-bib-0018] Pollack, C. E. , & Lynch, J. (2009). Health status of people undergoing foreclosure in the Philadelphia region. American Journal of Public Health, 99(10), 1833–1839. 10.2105/AJPH.2009.161380 19696373 PMC2741520

[hsc13573-bib-0020] Sareen, J. , Jagdeo, A. , Cox, B. J. , Clara, I. , ten Have, M. , Belik, S.‐L. , de Graaf, R. , & Stein, M. B. (2007). Perceived barriers to mental health service utilization in the United States, Ontario, and the Netherlands. Psychiatric Services, 58(3), 357–364. 10.1176/ps.2007.58.3.357 17325109

[hsc13573-bib-0021] Sweet, E. , Nandi, A. , Adam, E. K. , & McDade, T. W. (2013). The high price of debt: Household financial debt and its impact on mental and physical health. Social Science & Medicine, 91, 94–100. 10.1016/j.socscimed.2013.05.009 23849243 PMC3718010

[hsc13573-bib-0022] U.S. Department of Education . (2018). Collections. Accessed November 16, 2019. https://studentaid.ed.gov/sa/repay‐loans/default/collections

[hsc13573-bib-0023] U.S. Department of Education . (2019). Student loan delinquency and default. Accessed December 21, 2019. https://studentaid.ed.gov/sa/repay‐loans/default

[hsc13573-bib-0024] Walsemann, K. M. , Ailshire, J. A. , & Gee, G. C. (2016). Student loans and racial disparities in self‐reported sleep duration: Evidence from a nationally representative sample of USA young adults. Journal of Epidemiology and Community Health, 70(1), 42–48. 10.1136/jech-2015-205583 26254292

[hsc13573-bib-0025] Woolf, S. , & Aron, L. (2013). U.S. health in international perspective: Shorter lives, poorer health. National Academies Press. 24006554

